# Acceptability of a Conversational Agent–Led Digital Program for Anxiety: Mixed Methods Study of User Perspectives

**DOI:** 10.2196/76377

**Published:** 2025-11-04

**Authors:** Pearla Papiernik, Sylwia Dzula, Marta Zimanyi, Edward Millgate, Malika Bouazzaoui, Jessica Buttimer, Graham Warren, Elisa Cooper, Ana Catarino, Shaun Mehew, Emily Marshall, Valentin Tablan, Andrew D Blackwell, Clare E Palmer

**Affiliations:** 1 ieso Digital Health Cambridge United Kingdom

**Keywords:** mental health, anxiety, digital intervention, conversational agent, artificial intelligence, user experience, acceptability, engagement, thematic analysis

## Abstract

**Background:**

The prevalence of anxiety and depression is increasing globally, outpacing the capacity of traditional mental health services. Digital mental health interventions (DMHIs) provide a cost-effective alternative, but user engagement remains limited. Integrating artificial intelligence (AI)–powered conversational agents may enhance engagement and improve the user experience; however, with AI technology rapidly evolving, the acceptability of these solutions remains uncertain.

**Objective:**

This study aims to examine the acceptability, engagement, and usability of a conversational agent–led DMHI with human support for generalized anxiety by exploring patient expectations and experiences through a mixed methods approach.

**Methods:**

Participants (N=299) were offered a DMHI for up to 9 weeks and completed postintervention self-report measures of engagement (User Engagement Scale [UES]; n=190), usability (System Usability Scale [SUS]; n=203), and acceptability (Service User Technology Acceptability Questionnaire [SUTAQ]; n=203). To explore expectations and experiences with the program, a subsample of participants completed qualitative semistructured interviews before the intervention (n=21) and after the intervention (n=16), which were analyzed using inductive thematic analysis.

**Results:**

Participants rated the digital program as engaging (mean UES total score 3.7; 95% CI 3.5-3.8), rewarding (mean UES rewarding subscale 4.1; 95% CI 4.0-4.2), and easy to use (mean SUS total score 78.6; 95% CI 76.5-80.7). They were satisfied with the program and reported that it increased access to and enhanced their care (mean SUTAQ subscales 4.3-4.9; 95% CI 4.1-5.1). Insights from pre- and postintervention qualitative interviews highlighted 5 themes representing user needs important for acceptability: (1) accessible mental health support, in terms of availability and emotional approachability (Accessible Care); (2) practical and effective solutions leading to tangible improvements (Effective Solutions); (3) a personalized and tailored experience (Personal Experience); (4) guidance within a clear structure, while retaining control (Guided but in Control); and (5) a sense of support facilitated by human involvement (Feeling Supported). Overall, the DMHI met participant expectations, except for theme 3, as participants desired greater personalization and reported frustration when the conversational agent misunderstood them.

**Conclusions:**

Incorporating factors critical to patient acceptability into DMHIs is essential to maximize their global impact on mental health care. This study provides both quantitative and qualitative evidence for the acceptability of a structured, conversational agent–driven digital program with human support for adults experiencing generalized anxiety. The findings highlight the importance of design, clinical, and implementation factors in enhancing engagement and reveal opportunities for ongoing optimization and innovation. Scalable models with stratified human support and the safe integration of generative AI have the potential to transform patient experience and increase the real-world impact of conversational agent–led DMHIs.

**Trial Registration:**

ISRCTN Registry ISRCTN 52546704; https://www.isrctn.com/ISRCTN52546704

## Introduction

Demand for mental health care is increasing, with 1 in 8 individuals globally living with a mental health condition [1]. Despite this growing need, there are only 4 psychiatrists per 100,000 people worldwide [2], leading to long waiting lists and a lack of care [3-5]. The traditional 1:1 mental health care model is proving insufficient to meet the rising demand.

Digital mental health interventions (DMHIs) provide a cost-effective way to increase access to care worldwide [6], but face challenges with user engagement and adherence [7-9]. Engagement is often operationalized through objective behavioral metrics (frequency and duration of use), but users’ cognitive and emotional investment (subjective engagement), such as focused attention and psychological investment, is equally critical to understanding how users meaningfully engage with DMHIs to drive clinical impact [10,11]. Key factors impacting engagement include available guidance, perceived fit, and personalization [12]. Artificial intelligence (AI)–powered conversational agents, typically using tree-based dialogue systems, have shown potential to enhance DMHI engagement by offering more interactive and personalized experiences compared with self-guided activities [13]. Meta-analytic evidence suggests that these agents reduce attrition rates [14] and show promising efficacy [15-18]. However, the agent’s misunderstanding of user input can be frustrating [19]. Advances in generative AI, such as OpenAI’s ChatGPT (released November 2022), are rapidly evolving and enable more fluid and naturalistic interactions that have the potential to transform DMHIs. However, currently, most DMHIs rely on tree-based dialogue systems with templated responses to ensure a patient’s experience is safe, evidence-based, and deterministic. It is unclear how user familiarity with generative AI applications will impact perceived effectiveness and engagement with conversational-agent DMHIs reliant on tree-based dialogue systems.

Acceptability is central to developing and evaluating DMHIs as it is linked to engagement, effectiveness, and adoption [20]. Acceptability is a multidimensional construct that reflects how people think and feel about an intervention and whether they view it as appropriate, either before, during, or after using it [21]. Qualitative studies suggest that conversational agent–led DMHIs are perceived positively by users [22,23], in part by providing easily accessible and judgment-free environments [24]. However, uptake of such interventions is impacted by mixed expectations on their effectiveness [25,26]. Presence or absence of empathy, personalization, and the ability of the agent to understand user messages also impact the user experience [19,27]. Our current understanding of conversational agent–led DMHI acceptability relies on limited qualitative data (eg, app store reviews and answers to in-app free-text questions about positive and negative experiences) [15,28-31]. A deeper understanding of the many factors contributing to user perception and experience with conversational agent–led DMHIs is important to maximize their impact.

Using mixed methods is recommended when iteratively developing and evaluating complex interventions such as DMHIs [32,33]. Validated questionnaires offer standardized quantitative benchmarks for comparison with other interventions and evaluation of iterative improvements [34]. By contrast, qualitative methods, such as semistructured interviews, enable richer insights into context-specific user needs and intervention-specific experiences, and allow for the discovery of emergent topics [35]. Through open-ended questions and in-depth explorations of user perspectives and experiences, multidimensional concepts such as acceptability and subjective engagement can be assessed [32,35]. Combining these methods provides complementary insights, where quantitative data describe behavior across the sample, and qualitative findings clarify the underlying reasons and nuances of user experiences.

This study reports additional patient-reported end points from a propensity-matched study evaluating the effectiveness, acceptability, engagement, and safety of a digital program for adults with mild to severe symptoms of generalized anxiety [18]. Participants engaged with the program for up to 9 weeks via a smartphone app. The intervention consisted of a structured program using traditional and third-wave cognitive behavioral therapy (CBT) approaches delivered through multimedia content and text-based interactions with an AI-powered conversational agent. The conversational agent followed a tree-based dialogue system, using natural language processing to deliver appropriate clinician prewritten responses. The program was combined with human support, aimed to enhance user experience and adherence with the program, addressing documented barriers to engagement [12]. The digital program showed a mean reduction in anxiety symptoms comparable to traditional human-delivered therapy [18]. This substudy aimed to understand the acceptability, engagement, and usability of the digital program by combining quantitative and qualitative methods to explore patient expectations and experiences with the intervention and examine the extent to which the program met their needs. Self-report measures for subjective engagement, usability, and acceptability were collected postintervention to quantify the user experience, and qualitative semistructured interviews were conducted with a subsample of participants both before and after the intervention to provide deeper insights into user needs and experiences.

## Methods

### Study Design

This mixed methods substudy explored the perspectives and experiences of individuals with mild, moderate, and severe symptoms of generalized anxiety before and after engaging with a DMHI. It was conducted within a wider pragmatic, single-intervention arm study with a sample of 299 participants from the United Kingdom [18].

Potentially eligible individuals completed a clinical assessment with a qualified clinician to determine eligibility. At the point of consent, participants were asked if they wanted to participate in qualitative interviews. A subsample of participants (n=21) attended a semistructured interview preintervention. All participants were asked to download the software on their smartphone and engage with the 6-module program in their own time, over a 9-week period. Participants from the interviewed subsample were invited to attend a second semistructured interview after using the program. All outcomes collected at different time points are reported in Palmer et al [18]. For this study, engagement, usability, and acceptability measures, collected with validated questionnaires postintervention, were analyzed, as well as qualitative analysis of the pre- and postintervention semistructured interviews.

### Ethics Considerations

**Figure 1:**
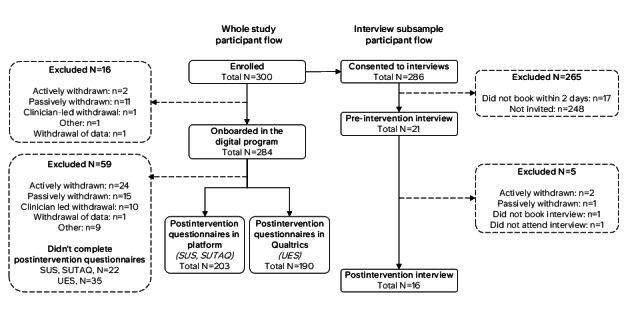
Participant flow for the study sample and interviewed subsample. Recruitment sources and preenrollment flow are described in Palmer et al [18]. Participants were withdrawn actively (requested withdrawal), passively (dropped out or disengaged), clinician-led (withdrawn on clinician recommendation), or for other reasons (eg, technical issues). Interview participation was offered on a first-come, first-served basis, with a 2-day response window, and invitations ceased once a sufficient number of preintervention interviews had been completed. Postintervention questionnaires were administered through ieso’s validated clinical delivery platform (System Usability Scale [SUS] and Service User Technology Acceptability Questionnaire [SUTAQ]) and through Qualtrics (User Engagement Scale [UES]).

The study was preregistered (ISRCTN ID 52546704) and obtained ethical approval before recruitment (IRAS ID: 327897, National Health Service [NHS] Research Ethics Committee: West of Scotland REC 4). The participant CONSORT (Consolidated Standards of Reporting Trials) flowchart for the whole sample and the interviewed subsample is summarized in Figure 1. For more details on participant flow before enrollment, please refer to Palmer et al [18]. In line with the Declaration of Helsinki, informed consent was obtained from all participants before enrollment, including optional consent for participation in semistructured interviews. Participants were debriefed at the conclusion of the study.

### Description of Digital Intervention

The intervention consisted of a 6-module digital program delivered via a smartphone app (ieso Digital Program; software name IDH-DP2-001), developed by ieso Digital Health [36], a mental health technology company and provider within NHS Talking Therapies delivering 1:1 human-delivered CBT via a typed modality to treat patients with common mental health disorders. The ieso Digital Program used a conversational agent to guide users through activities, with clinical oversight and user support provided by nonclinical research coordinators. The program was designed for people primarily presenting with symptoms of generalized anxiety and was based on principles from traditional CBT and third-wave approaches such as acceptance and commitment therapy. The 6 modules consisted of an introduction module, 3 core modules, and 2 consolidation modules. All modules began with a symptom “check-in” consisting of the 7-item Generalized Anxiety Disorder (GAD-7) scale and 9-item Patient Health Questionnaire (PHQ-9) scale. The minimum meaningful program dose was defined a priori by accredited CBT therapists as completion of modules 1-3 and the module 4 check-in. There were 16 sessions total, released on a timed schedule, subject to completing the prior session, allowing space for practice and reflection between sessions. Each session included videos, educational content, conversations, and worksheets written by accredited clinicians. The clinical team had an average of 14 years’ experience delivering therapy to patients with complex needs and diverse backgrounds in the United Kingdom. To ensure safety and clinical quality of the messages delivered to the user, the conversational agent used in this version of the program followed a tree-based dialogue system, using natural language processing to understand user input and deliver appropriate prewritten responses. The program complied with ISO 13485 [37] and was registered as a UKCA (UK Conformity Assessed) marked Class 1 medical device. For additional details, including screenshots of the interface and schematics of the program, see Palmer et al [18].

### Participants

Adults with mild to severe anxiety symptoms were recruited between October 10, 2023, and February 2, 2024, through 3 streams: (1) referrals to ieso’s typed therapy service (self-referral or via their NHS Provider) [38]; (2) responses to online advertisements; and (3) responses to email invitations through the NIHR BioResource [39].

Participants had to meet the following eligibility criteria: (1) be over the age of 18 years at point of recruitment; (2) GAD-7 total score >7; (3) PHQ-9 total score <16; (4) have a primary presenting problem of generalized anxiety disorder, as established through a clinician assessment (based on the International Classification of Diseases, 10th Revision [ICD-10] code in line with the NHS Talking Therapies manual [40]); (5) have access to a smartphone and internet connection; and (6) be registered with a general practitioner in the United Kingdom. To be suitable for the CBT protocol used in the intervention, individuals were excluded if presenting with (1) complex comorbid presentations (diagnosis of multiple disorders, psychotic or personality disorder, autism spectrum condition, or intellectual disability); (2) untreated mental health conditions (including substance misuse), except generalized anxiety disorder or major depressive disorder; or diagnoses of (3) posttraumatic stress disorder, obsessive-compulsive disorder or panic disorder. Additionally, individuals were excluded if they (1) were currently receiving psychological therapy, (2) had changes in psychiatric medication in the past 1 month; or (3) displayed a significant risk of harm to self, to others, or from others (established with the clinical assessment). Any individual who had previously participated in user research for the digital program was also excluded.

Considering the study’s aims and design, participant specificity, and experienced user researchers collecting data, a moderate sample size between 15 and 30 participants was estimated to provide sufficient informational power [41]. We aimed to enroll up to 30 participants to account for an estimated 50% attrition rate with the goal of having at least 15 postintervention interviews. Those who consented to interviews self-selected into the interviewed subsample by signing up for a preintervention interview slot on a first-come, first-served basis. If they did not sign up within 2 days of receiving the invitation, they were sent the information to continue with the app download to avoid delays. Interview invitations were sent out from December 2023, 2 months into the enrollment period. Interview invitations were stopped once a sufficient sample had attended a preintervention interview.

Vouchers up to a total of £60 (US $81.1) were provided for participating in the study based on study assessments and completion of modules. An additional £15 (US $20.2) voucher per interview was provided to those who attended an interview.

### Patient and Public Involvement

Experts by lived experience were involved as members of a patient and public involvement (PPI) panel. PPI members took part in study conceptualization and supported recruitment through co-designing marketing campaigns and participant-facing documents. PPI members were also involved at the end of the thematic analysis process through a workshop aimed to discuss the coherence of thematic groupings and theme names, and limitations and implications of these results. Before the workshop, PPI members were introduced to thematic analysis and qualitative methods. More details are provided in Multimedia Appendix 1.

### Data Collection and Analysis

#### Self-Report Measures of User Experience

##### Overview

Validated self-report measures of user experience were collected at postintervention between January and April 2024 (after completion or after 9 weeks), via either ieso’s validated clinical delivery platform (System Usability Scale [SUS] and Service User Technology Acceptability Questionnaire [SUTAQ]) or Qualtrics (User Engagement Scale [UES]). Demographic data were collected at enrollment and are summarized in Table 1.

**Table 1:** Sample characteristics of all study samples.

Demographic and category		Questionnaire sample (N=203)	Interview subsample (n=21)	
			Preintervention sample (n=21)	Postintervention sample (n=16)
Baseline GAD-7 score, mean (SD)^a^		12.4 (3.4)	11.8 (3.2)	11.6 (3.6)
Baseline PHQ-9 score, mean (SD)^b^		7.9 (3.8)	7.7 (2.4)	7.7 (2.5)
Age, mean (SD)		41.0 (12.0)	46.0 (13.4)	44.0 (13.3)
**Gender, n (%)**				
	Female	163 (80.3)	17 (81.0)	13 (81.3)
	Male	34 (16.7)	3 (14.3)	2 (12.5)
	Other	2 (1.0)	1 (4.8)	1 (6.3)
	Not known	4 (2.0)	0 (0)	0 (0)
**Ethnicity, n (%)**				
	White	185 (91.1)	19 (90.5)	15 (93.8)
	Mixed	3 (1.5)	0 (0)	0 (0)
	Asian	9 (4.4)	2 (9.5)	1 (6.3)
	Black/African/Caribbean/Black British	1 (0.5)	0 (0)	0 (0)
	Other	1 (0.5)	0 (0)	0 (0)
	Prefer not to say	4 (2.0)	0 (0)	0 (0)
**Long-term condition, n (%)**				
	Yes	83 (40.9)	9 (42.9)	6 (37.5)
	No	112 (55.2)	10 (47.6)	9 (56.3)
	Not known	8 (3.9)	2 (9.5)	1 (6.3)

^a^7-item Generalized Anxiety Disorder categories: mild (scores 5-9), moderate (scores 10-14), and severe (scores ≥15).

^b^9-item Patient Health Questionnaire categories: mild (scores 5-9), moderate (scores 10-14), moderately severe (scores 15-19), and severe (scores ≥20).

##### Engagement

Engagement was evaluated with the UES short form [42], a validated scale to measure user engagement with digital systems. This scale consisted of 12 items across 4 subscales: (1) *Focused Attention* (ie, how absorbed the individual was in the intervention); (2) *Perceived Usability* (ie, whether the intervention was taxing or frustrating to use); (3) *Aesthetic Appeal* (ie, how visually appealing the intervention was; and (4) *Rewarding* (ie, whether using the intervention was worthwhile). The 12 items were rated on a 5-point scale (1=strongly disagree to 5=strongly agree). A high score can be interpreted as positive perceived engagement. Items were presented in a random order for each participant. Three items on the *Perceived Usability* subscale were reverse scored, such that a lower value on each item was associated with greater perceived engagement. Individual items are reported as the number and percentage of participants who selected each Likert rating. These were averaged across items within each subscale to estimate the distribution of participants who on average agreed or disagreed with the items within each subscale. Average percentage agreement or disagreement to subscales across participants was also reported, calculated by regrouping all levels of agreement (agree/strongly agree) or disagreement (disagree/strongly disagree).

##### Usability

Usability was evaluated with the SUS [43]. The SUS consisted of 10 items relating to the overall usability of the intervention. Items were rated on a 5-point scale (1=strongly disagree to 5=strongly agree). An overall SUS score was calculated as a sum across all items after reverse coding certain items and scaling to produce a score from 0 to 100 [43]. A high score can be interpreted as positive perceived usability. Individual items are reported as the number and percentage of participants that selected each Likert rating. These were averaged across all items to estimate the proportion of participants that on average agreed or disagreed with items across the questionnaire. Average percentage agreement or disagreement per item and for the whole questionnaire across participants was also reported, calculated by regrouping all levels of agreement (agree/strongly agree) or disagreement (disagree/strongly disagree).

##### Acceptability

Acceptability was quantitatively evaluated with the SUTAQ [44]. It consisted of 22 items across 6 subscales: (1) *Care Personnel Concerns* (ie, concerns about the skills and continuity of the personnel looking after them); (2) *Enhanced Care* (ie, beliefs that the intervention can improve care); (3) *Increased Accessibility* (ie, beliefs that the intervention enhanced access to care); (4) *Kit as Substitution* (ie, beliefs that the intervention may be an alternative to regular care); (5) *Privacy and Discomfort* (ie, concerns privacy and the intervention interfering with their daily life or making them feel uncomfortable); and (6) *Satisfaction* (ie, beliefs indicating acceptance and satisfaction with the kit and service provided). Participants rated their level of agreement with each item on a 6-point Likert scale (1=strongly disagree to 6=strongly agree). High scores can be interpreted as positive user perception, except for subscales *Care Personnel Concerns* and *Privacy and Discomfort* that are inverted; therefore, a low score is interpreted as a positive user perception. The intermediate value 3.5 is interpreted as a point of neutrality. Reverse coding was carried out on 1 item in the *Kit as Substitution* subscale [44] (item 18; see Table S3 in Multimedia Appendix 2). Individual items are reported as the number and percentage of participants that selected each Likert rating. These were averaged across items within each subscale to estimate the distribution of participants agreeing or disagreeing with the items within each subscale. Average percentage agreement or disagreement to subscales across participants was also reported in text, calculated by regrouping all levels of agreement (mildly, moderately, and strongly agree) or disagreement (mildly, moderately, and strongly disagree).

### Qualitative Data

Semistructured interviews pre- and postintervention provided qualitative data about user expectations and experiences with the program, to assess the acceptability of the ieso Digital Program in meeting the needs of participants. Preintervention interviews, conducted by 2 user researchers between December 2023 and February 2024 (SD and MZ), lasted 16-38 minutes (mean 23 minutes). Postintervention interviews, conducted by 1 user researcher between February and April 2024 (MZ), lasted 30-47 minutes (mean 39 minutes). Interview topic guides were developed by user researchers, based on learnings from literature and from extensive internal user research and PPI workshops during the development phase of the program. Topic guides were reviewed by internal stakeholders with clinical and product expertise. Preintervention interviews explored participants’ motivations to take part, their experience and views of using technology for mental health, and their expectations for the intervention. Postintervention interviews explored participants’ overall experience with the intervention, engagement barriers and facilitators, perceived usefulness and effectiveness, and perceived safety and support. Refer to Multimedia Appendix 1 for detailed interview schedules. Interviews were conducted on Microsoft Teams and were automatically transcribed on Dovetail [45] and reviewed and anonymized by researchers.

Pre- and postintervention interviews were analyzed using reflexive thematic analysis [46,47], led by a researcher trained in qualitative research methods (PP) who familiarized with the data by reading interview transcripts multiple times and completed line-by-line inductive coding at both the semantic and latent levels in Dovetail. Codes were then grouped into candidate themes, which were then reviewed by returning to the underlying data. Final themes were defined by clarifying thematic boundaries and writing synopses. To ensure reflexivity and rigor in interpretations, analysis was regularly discussed between researchers (PP, CEP, MB, and MZ). A workshop with individuals with lived experience of mental health was held to review and discuss the thematic analysis findings. Final themes grouped both pre- and postinterview data linked by the common underlying concept of the theme. Results describe expectations and experiences in each theme. A detailed description of the thematic analysis process and workshop is available in Multimedia Appendix 1.

The framework method [48,49] was used to explore patterns between preintervention expectations and postintervention experiences. Inductive themes from the thematic analysis were used to create an analytical framework, and coded extracts were categorized within it. A framework matrix summarized data for each participant and theme by interview time (pre and post), with illustrative quotes. The framework matrix was interpreted by exploring patterns within and between participants, before and after the intervention, and relevant nuances and insights were added to our description of themes developed through thematic analysis.

## Results

### Self-Report Measures of User Experience

#### Overview

**Figure 2:**
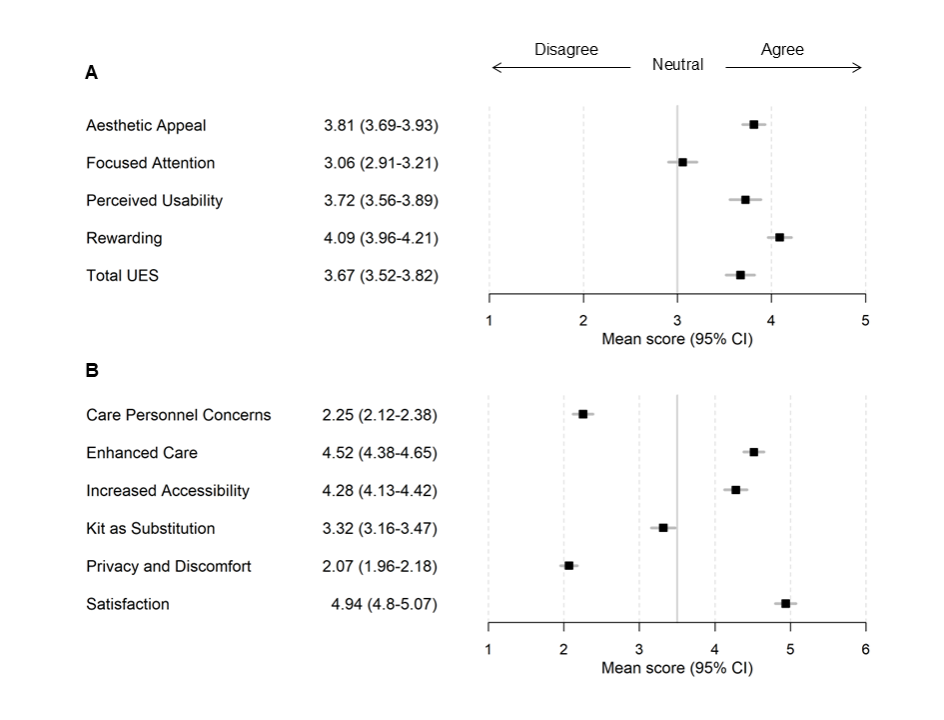
Self-reported user experience mean scores with 95% CIs. (A) User Engagement Scale (UES) subscales and total score. (B) Service User Technology Acceptability Questionnaire (SUTAQ) subscales. Higher scores on all subscales indicate more positive user sentiment, except for the SUTAQ *Care Personnel Concerns* and *Privacy and Discomfort* subscales, where lower scores indicate more positive sentiment.

The final sample for analysis included 203 participants who completed the SUS and the SUTAQ, and 190 participants who completed the UES. Table 1 provides an overview of the demographic and clinical characteristics of participants enrolled in the intervention who completed the questionnaires (N=203). Of these, 163 (80.3%) were female, with a mean age of 41 (SD 12.0; range 19-75) years, and 168 (82.8%) completed the minimum meaningful clinical dose [18]. UES and SUTAQ subscale scores are reported in Figure 2, and average agreement or disagreement across scale items are reported in Tables 2 (UES and SUS) and 3 (SUTAQ). Item-level data for each scale can be found in Tables S1-S3 in Multimedia Appendix 2.

**Table 2:** Agreement and disagreement with the self-reported measures of engagement (UES^a^) and usability (SUS^b^).

Scale			Mean proportion agreement across all items or items within each subscale, %								
			1=Strongly disagree		2=Disagree		3=Neither agree nor disagree		4=Agree		5=Strongly agree
**UES (n=190)**											
	Aesthetic Appeal	1.2		4.6		24.2		51.8		18.3	
	Focused Attention	6.5		26.3		29.3		30.9		7.0	
	Perceived Usability	4.4		14.2		13.5		40.5		27.4	
	Rewarding	1.9		3.7		10.5		51.4		32.5	
	Total	3.5		12.2		19.4		43.6		21.3	
**SUS (n=203)**											
	Total	1.4		5.3		9.7		44.6		39.0	

^a^UES: User Engagement Scale.

^b^SUS: System Usability Scale.

**Table 3:** Agreement and disagreement with the self-reported measure of acceptability (SUTAQ^a^).

Scale		Mean proportion agreement across all items or items within each subscale, %						
		1=Strongly disagree	2=Moderately disagree	3=Mildly disagree	4=Mildly agree	5=Moderately agree	6=Strongly agree	
**SUTAQ (N=203)**								
	Care Personnel Concerns	36.1	27.4	19.1	11.2	4.9	1.3	
	Enhanced Care	4.8	5.2	9.1	25.3	25.5	30.1	
	Increased Accessibility	5.8	6.4	12.8	28.1	23.2	23.8	
	Kit as Substitution	14.8	16.9	21.0	24.1	15.6	7.6	
	Privacy and Discomfort	45.2	23.8	15.8	10.8	3.3	1.1	
	Satisfaction	2.6	2.8	6.1	16.3	31.4	40.9	

^a^SUTAQ: Service User Technology Acceptability Questionnaire.

#### Engagement

Overall, the mean total score for the UES was 3.7 (95% CI 3.5-3.8), indicating agreement that the program was engaging. On average, across the intervention sample who completed the UES (n=190), 159 (83.7%) agreed that the program was rewarding and worthwhile (*Rewarding*: mean 4.1; 95% CI 4.0-4.2), 129 (67.9%) perceived the app not to be frustrating or taxing (*Perceived Usability*: mean 3.7; 95% CI 3.6-3.9) and 133 (70%) found the app aesthetically pleasing (*Aesthetic Appeal*: mean 3.8; 95% CI 3.7-3.9). There were mixed views on whether participants were fully focused on the experience (*Focused Attention*: mean 3.1; 95% CI 2.9-3.2). Item-level scores indicated participants generally disagreed that they were “getting lost” in the experience, and there were mixed views on whether participants were fully absorbed in the app (see Table S1 in Multimedia Appendix 2).

#### Usability

Self-reported usability scores were high with a mean total score for the SUS of 78.6 (n=203; 95% CI 76.5-80.7). Item-level data (Table S3 in Multimedia Appendix 2) show that across the intervention sample (n=203), most participants agreed that the system was quick to learn to use (n=185, 91.1%), without the support of a technical person (n=188, 92.6%); 180 (88.7%) felt confident using the app and 179 (88.2%) found it easy to use. The item participants agreed the least strongly with was whether they would like to use the system frequently (n=135, 66.5% agreement).

#### Acceptability

Across the intervention sample (n=203), 180 (88.7%) participants were satisfied with the intervention (*Satisfaction*: mean 4.9; 95% CI 4.8-5.1). On average, 164 (80.8%) participants found that the app could improve care (*Enhanced Care*: mean 4.5; 95% CI 4.4-4.7) and 152 (74.9%) found that it increased access to care (*Increased Accessibility*: mean 4.3; 95% CI 4.1-4.4), without interfering with their daily life or making them feel uncomfortable (*Privacy and Discomfort*: mean[reverse scored] 2.1; 95% CI 2.0-2.2), and without concern about the skills and continuity of the personnel looking after them (*Care Personnel Concerns*: mean[reverse scored] 2.3; 95% CI 2.1-2.4). There were mixed responses about whether the program could be used as a substitution for their regular mental health care (*Kit as Substitution*: mean 3.3; 95% CI 3.2-3.5).

### Qualitative Findings

#### Overview

Table 1 provides an overview of the demographic and clinical characteristics of participants enrolled in the interview subsample (n=21). Participants in the interview subsample were mostly White (n=19, 90%) and women (n=17, 81%), with a mean age of 46 (SD 13.1; range 19-72) years. Half of the sample had moderate anxiety (n=10, 48%), followed by mild (n=7, 33%) and severe (n=4, 19%) anxiety. Five participants did not do postintervention interviews: 3 were withdrawn from the study, and 2 did not book or attend their postintervention interview. Participants were highly engaged, with 14 out of 21 (67%) of the pre-intervention and 12 out of 16 (75%) of the postintervention interview subsamples completing a minimum meaningful clinical dose of the program.

**Figure 3:**
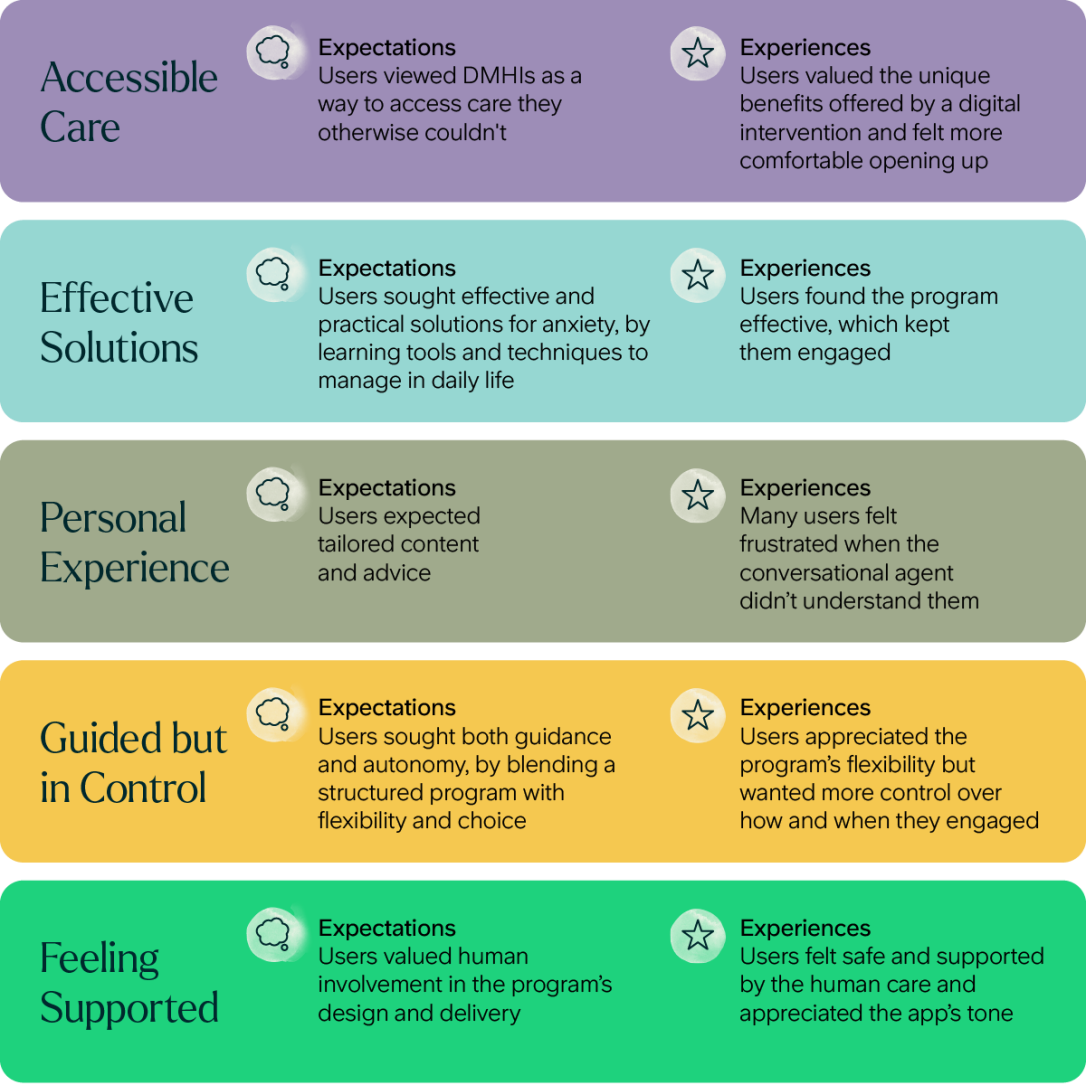
Thematic map. Five qualitative themes illustrate user needs that influence the acceptability of conversational agent–led digital mental health interventions (DMHIs). Each theme summarizes user expectations and experiences drawn from pre- and postintervention interviews.

Figure 3 shows a map of the themes developed through thematic analysis of the pre- and postintervention interviews. Five themes represent patient needs for conversational agent–led DMHIs and describe to what extent they were met, based on their expectations and experiences with the ieso Digital Program: (1) Accessible Care, (2) Effective Solutions, (3) Personal Experience, (4) Guided but in Control, and (5) Feeling Supported. Quotes are provided with interview type (pre, post), participant gender, age, and baseline anxiety severity.

#### Theme 1: Accessible Care

This theme describes how participants sought mental health support that was accessible in terms of both availability and emotional approachability. The digital format was perceived as offering this kind of access and reached users who would not have accessed support otherwise.

Many participants were motivated to access mental health support that they felt was difficult to obtain through the NHS. Participants appreciated that the program was a faster, easier, and more affordable way to access support.

It's always easy to get an appointment but then from there the support is pretty much nonexistent....Basically, the advice is you could probably do with some more talking therapy, you can go on the waiting list and that will be up to a year, otherwise you can pay. So that's why the program is such a positive thing, because otherwise there's nothing.

post, P14, female, 53, severe anxiety

Some participants avoided seeking support due to guilt over using NHS resources. For some, the digital program was perceived as a good “first point of call” to address mental health concerns, and other options could be available after if needed.

I see it like, if I lined up Domino's, this is like Domino number one.

pre, P16, female, 44, severe anxiety

For many participants, a digital modality was an advantage. Participants wanted to reflect on their experiences and understand their triggers, thoughts, and feelings, and some believed that a digital program was better suited for them to do this work. Indeed, several participants expected and found it easier to express their feelings to an app compared with a human therapist, especially if they had prior negative experiences with therapy. Participants felt less judged or embarrassed to open-up, and therefore self-censored less. Some participants also felt that the program may be less biased than a human therapist.

I liked it because I felt there wasn't any sense of judgment there. I've had a bad experience with therapy before where I didn't really get along with the therapist and there was quite a lot of judgment passed...And just knowing that that wasn't even something that could happen was really reassuring.

post, P4, female, 19, mild anxiety

Several participants preferred the privacy DMHIs would provide, compared with online therapy calls that could be overheard, and valued having more time to understand information and reflect before answering.

Text based certainly for me, I'm able to be much more thoughtful, much more introspective, much more precise is my answer when they're written rather than spoken. I think it gives me time to think deeper about things.

pre, P12, female, 48, moderate anxiety

Participants also valued being able to revisit and reflect on past content and discussions saved and centralized on an app. Many wanted continued app access poststudy to further review content.

If I could go back into the app to see how I wrote down what I was feeling, that would be, that would really reinforce it all.

post, P9, female, 57, mild anxiety

#### Theme 2: Effective Solutions

This theme describes participants’ motivation to access effective solutions for anxiety. They sought practical tools to manage anxiety and would continue engaging if they perceived the program as effective.

A key motivation expressed in pre- and postintervention interviews was to access a solution-focused intervention to effectively manage or reduce anxiety. Participants differentiated the need to “offload,” from their current need to “change behavior” and receive “tools” and “solutions.” Most wanted to learn techniques to manage their anxiety long-term, while some sought momentary support or both. Participants practiced and applied techniques in everyday life, particularly when anxious or when facing a stressful situation.

[When I’m in a triggering situation], I would go either directly back to the app or I would go back to my book and I would look at what I've learned and I would go, ok, how can I approach this situation differently?

post, P16, female, 44, severe anxiety

Accessing helpful content and learning new information and techniques was crucial to participants and was for many a key motivator, even for participants who experienced frustration when the conversational agent misunderstood them. Nearly all participants interviewed found the program useful and effective. Seeing improvements and perceiving the program as beneficial was an important motivator for continued engagement with the program and continued application of techniques learned.

I was motivated to continue because each module had something which helped me. I felt that it was helping me reduce my anxiety right from the beginning. So, you wanted to do the next module.

post, P5, female, 68, mild anxiety

Participants noted the program’s time and effort demands but emphasized the importance of engaging to see benefits. For many, this was acceptable as they began the program highly motivated and ready to engage; however, 1 participant was surprised by the program’s workload and another shared that they could not have engaged similarly at another time in their mental health journey.

If you can stick with it and really commit to trying the things it suggests you try, you'll definitely find it helpful....But you have got to be willing to kind of commit to it and put in the time and do what it's asking you to do. So, you've got to be in the right headspace.

post, P14, female, 53, severe anxiety

#### Theme 3: Personal Experience

The third theme describes how participants wanted the program to be tailored and feel personal, but sometimes found the conversational agent to be generic or unnatural, and often felt misunderstood by it.

Preintervention, several participants expected personalized strategies for anxiety, tailored advice, and pathways or exercises based on their input or assessments. Half of the participants viewed technology and an AI guide as an opportunity for tailored and accurate support.

I would assume that based on my answer to questions or how I'm responding to things, that machine learning will tell the digital guide move things to get onto the right journey.

pre, P7, female, 53, moderate anxiety

Postintervention, some found the conversational agent impersonal or generic, and desired more context-specific advice, historical knowledge use, and the ability to ask questions.

I didn't quite enjoy that I was talking to a robot that didn't tell me things tailored to my needs, only generic things. So many times it didn't really matter what I said, the robot only repeated what I said.

post, P2, male, 33, moderate anxiety

Feeling heard and understood was crucial to participants. In preintervention interviews, interacting with a conversational agent was viewed as acceptable if it was well-trained, lacked bias, and was able to understand them and offer thoughtful answers.

As long as it's been well trained, it will be interesting to see what it comes back with. That it’s not just a sort of trite responses you get when you're trying to get customer service out.

pre, P12, female, 48, moderate anxiety

Postintervention, some participants explained that the downside of a conversational agent was that they could not receive empathy and be understood like they would with a therapist. Nearly all users reported instances where the conversational agent wrongly paraphrased messages or misunderstood complex thoughts and feelings. This often led to frustration or confusion. Safety messages that appeared when the user did not feel they were needed could lead to frustration and distraction from the session.

I think there was a lot of instances where it was clear that whatever I typed it hadn’t understood. In a way that’s like sitting in a room with a therapist and you tell them something and they just look blank.

post, P15, nonbinary, 42, severe anxiety

Some participants found the conversational agent’s understanding abilities were less bothersome and less present over time, and some explained that they learned to communicate more effectively with it.

I think as the program went on, I got to know the program and I got maybe a bit better myself at finding ways to word things.

post, P14, female, 53, severe anxiety

However, others explained that simplifying their answers to ensure the conversational agent would understand impacted their ability to express their emotions.

And so you learn to keep it really simple. But that sometimes didn't help, I couldn't really express how I was feeling because the chatbot didn't understand.

post, P5, female, 68, mild anxiety

Several participants were not too impacted by these limitations, finding them expected and acceptable for a conversational agent, and not interfering with the overall program’s usefulness.

You could tell it was looking for specific responses, but that's fine. That's what they do.

post, P2, male, 33, moderate anxiety

Postintervention, participants had mixed views on the conversational agent’s human-likeness, some finding it human-like and others robotic. Notably, 2 participants valued its neutrality and “ambiguity,” neither distinctly human nor robotic, and neither male nor female. However, conversations feeling robotic and preformatted answers could feel restrictive and frustrating, and affected participant’s sense of agency, an important need discussed in the next theme.

I do think some of the time when you only have one response to give, sometimes I wanted to have choice or say my own thing. If you read back through my data, sometimes you can probably see I'm getting a bit frustrated, if you want to say more sometimes and then you're not given that option. And that sort of forces you out of your own voice and into the voice of whoever programmed it.

post, P12, female, 48, moderate anxiety

#### Theme 4: Guided but in Control

The fourth theme highlights participants’ desire to have autonomy and control over how they engaged with the program while also wanting structure and guidance throughout. Being self-directed within a flexible yet structured intervention was valued by participants and was a motivation to participate in the study.

Accessing a time-bound and structured program through an app was seen by participants as useful for personal accountability to help them regularly engage in the work they wanted to do to feel better.

I think this would be helpful because I can tap in and out of it as and when. And it's something that’s available, the app is always there for you to use. And at the same time there's time frames on it. So, you have to do things by certain dates. So there's a bit of a routine, but also a bit of flexibility.

pre, P1, female, 35, mild anxiety

Reminders were also seen as important to support adherence to the program, particularly considering reported engagement challenges such as their mental health, forgetfulness, or external obligations.

The reminders did serve, as a prompt to say, OK, just reminding you that you got your next session and you’ll be on track to complete the study.

post, P8, male, 32, mild anxiety

Accessing a program that is easy to navigate, with a “logical pathway” to follow, was important. Participants also wanted to be guided toward what they should practice and reflect on, through conversational agent prompts and exercises. One participant viewed conversational agents as acceptable only if used within a structured program.

The other app I used, I would say something and then the chatbot would reflect that back to me. I'm assuming there is a structured program in the ieso app. So, it's not just going to be stuff I say being reflected back, there would be ideas for me and something for me to follow.

pre, P13, female, 55, moderate anxiety

In postintervention interviews, some participants expressed that having more in-app features and sessions to support practice and review content would be helpful, as well as guided support when experiencing symptoms of anxiety, such as meditation or breathing exercises.

I think to actually have some, a few recorded meditations that people could turn to relax breathing techniques or relaxation, especially if you're having a bit of a crisis and you need to calm down.

post, P13, female, 55, moderate anxiety

In parallel to wanting guidance and structure, autonomy was important to participants.

Nothing feels forced upon you. Even the reminders I think are gentle, they’re like a little nudge rather than a big old slap.

post, P16, female, 44, severe anxiety

Many participants expressed high satisfaction with the intervention’s flexibility and ability to fit into their lives, allowing engagement at their own pace, when in the right headspace, and in various settings. Several participants, therefore, saw the program as particularly adapted for people with demanding jobs or caregiving responsibilities.

I don't have a lot of time to attend scheduled appointments. So being able to pick something up to help my own mental health in my own time is more likely to be something I would use in the future than in-person therapy.

post, P6, female, 44, moderate anxiety

Many participants desired more control over session timing, duration, and frequency. While some liked time locks between sessions to practice, others found them either too far apart, such that they hindered progress or timely support, or too close together, leaving insufficient time to practice.

Having to wait a few more days until the next session, I understand why. But sometimes I was really into a mood where I needed those sessions. And after a few days when the session was available, I wasn't available myself.

post, P2, male, 33, moderate anxiety

I thought early on it was the sessions are slightly too close together and I thought I didn't always have time to practice.

post, P10, female, 30, mild anxiety

#### Theme 5: Feeling Supported

The final theme describes how participants found the program to be supportive and safe, facilitated by human support.

Clinician involvement in the design of the program provided credibility. Participants were willing to engage as the content was clinically backed and written by humans.

I think if it's controlled, it's preprogramed responses written by an expert then, I have a lot of confidence in that system.

pre, P3, female, 31, severe anxiety

If users experienced increased anxiety during the study, it was attributed to external stressors and users were able to speak to a clinician. Some users said that negative emotions could sometimes arise, but this was viewed as expected when engaging in such exercises.

There were a few moments where it would ask you to think of something negative to practice. But I think that's how those sessions work. And at the end of it, I always felt better. But yeah, there was the odd moment, when it was getting you to think something, but I can't see a way for it to get you to practice and explain it without that.

post, P10, female, 30, mild anxiety

Participants valued human support throughout the program.

I think it was good that you can just pick it up and do it as and when, while also having some support from real people if and when you needed that.

post, P1, female, 35, mild anxiety

Regular calls with research coordinators were beneficial for adherence and engagement with the program, providing gentle reminders or deadlines in addition to app reminders.

It helped with accountability knowing that someone was going to call because then if I hadn't done it, that wouldn't be good.

post, P10, female, 30, mild anxiety

Importantly, regular check-ins with research coordinators and the option to consult a clinician provided users with a sense of safety and reassurance during the program, and knowing about this human element was a motivating factor to take part in the study. Knowing they could talk to a human was important even when participants did not experience the need for it. Several participants suggested integrating easy ways to contact a human within the app.

That was the good thing about those calls [with the research coordinator], you did know there was actually somebody there. I never had to contact a clinician or anything, but it was always very clear that that is an option if that is necessary.

post, P4, female, 19, mild anxiety

While most felt supported by the human contact provided, a few desired more frequent clinician interaction and guidance. Some accessed the human support they needed outside of the program, through friends and family, and 1 participant explained that the program itself encouraged and helped them seek social support.

Postintervention, participants expressed that the program’s design and content created a supportive and encouraging environment. Several participants described the program as “calming,” “relaxing,” and “gentle”; the content as “lovely” and “therapeutic”; and the conversational agent as “friendly” and “reassuring.”

And the acknowledgment at the end, “Let's acknowledge that this may be hard for you,” “Are you willing to commit to it?”. At that level of acknowledgement, you feel heard. It was like having a cheerleader on your side, you know? That was nice I thought.

post, P12, female, 48, moderate anxiety

## Discussion

### Principal Findings

This mixed methods study demonstrates the overall acceptability of a digital program driven by an AI conversational agent with human support for adults experiencing generalized anxiety symptoms. Quantitative self-reported measures showed that participants found the digital program rewarding and easy to use, and that it both increased access to and enhanced their mental health care. Qualitative interviews provided an in-depth understanding of factors important for DMHI acceptability, giving context to the quantitative findings—such as guiding users, fostering a sense of support, and observing tangible mental health improvements. However, participants frequently reported a lack of personalization and frustrations with the conversational agent’s misunderstandings, and there were mixed views on whether it could serve as a substitute for standard care. Using mixed methods enabled a deeper understanding of patient acceptability and offered a new perspective on previously reported outcomes [18], capturing participants’ affective and cognitive investment beyond usage metrics [50]. These findings highlight opportunities to optimize and innovate DMHIs to enhance their acceptability and impact on mental health care.

In this study, many participants sought a self-directed and autonomous solution while also wanting to feel supported, highlighting the need to balance structure and guidance with personal agency in DMHIs. Users appreciated the structured program and wanted more in-app support, such as prompts or worksheets to facilitate reflection and practice. By contrast, some participants wanted increased control over their pace through the program, echoing findings from other conversational agent DMHIs [51]. For example, although time-locked sessions were designed to encourage practice between sessions, many participants preferred having greater control over this. Most participants found the program easy to use, as reflected in a high mean SUS score of 78.6, which exceeds scores reported for other conversational agent DMHIs (63.6-66.2) [19]. The program also met participants’ expectations regarding access and flexibility, with 152 out of 203 (74.9%) agreeing that it increased access to care (SUTAQ *Increased Accessibility* subscale).

Despite wanting a self-led digital solution—perceived by some participants as a judgment-free and less intimidating way to access mental health support without talking to a therapist—human support remained crucial for building trust in the program, ensuring safety, and maintaining engagement. Nonclinical research coordinators, who provided motivational support through emails and phone calls, were perceived as helpful in fostering a sense of accountability. Participants also valued having the option to talk to a clinician if needed. This aligns with patient perceptions that DMHIs should involve clinicians and facilitate access to mental health professionals [23,26]. Moreover, this is consistent with findings that DMHIs with human support have lower dropout rates [14] and better outcomes [52], particularly at higher symptom severity [18]. Most participants reported minimal concerns about personnel skills and continuity of care (SUTAQ *Care Personnel Concerns* subscale; mean 2.3, range 1-6), highlighting the acceptability of this support model for real-world implementation. Nonetheless, there were mixed views on the acceptability of the digital program as a replacement for regular mental health care (SUTAQ *Kit as Substitution* subscale; mean 3.3, range 1-6). As individual needs and preferences influence the acceptability of DMHIs, it is essential to ensure that people are offered an informed choice in how they receive care. Moreover, although human support can enhance acceptability, implementations must remain scalable to meet the rising demand for mental health care. In the current program, comparable outcomes to human-delivered care were achieved with up to 8 times fewer clinician hours [18]. Innovative implementation approaches should be explored to further optimize the scalability, acceptability, and engagement of DMHIs that incorporate human support [53,54].

Acceptability can be further enhanced by improving the conversational agent. Being misunderstood by the agent was a significant source of frustration in this study and is a common limitation of tree-based dialogue systems [15,16,51,55,56]. Recent advances in generative AI have transformed human-technology interaction by enabling dialogue systems that emulate fluent human conversation, making users feel heard and understood [57]. Users have reported finding rule-based mental health apps less satisfying than those using generative conversations [58], and recent findings suggest that generative conversational agents may have a greater ameliorative impact on psychological distress than retrieval-based ones [59]. While further research is needed, these preliminary results indicate that using generative AI could enhance engagement and effectiveness in DMHIs. However, generative AI also introduces new patient risks [60,61]. Its integration into DMHIs should therefore be guided by mental health professionals [62], with rigorous frameworks in place to evaluate clinical risk and quality in mental health contexts [63].

Interestingly, despite the conversational agent’s limitations, many participants remained engaged because of the content’s usefulness. This is consistent with prior research linking perceived usefulness to greater engagement [12] and to the value users place on insights gained from DMHIs [15,56]. In this study, 180 out of 203 (88.7%) participants found the intervention satisfactory (180/203; SUTAQ *Satisfaction* subscale), 164 out of 203 (80.8%) reported that it enhanced care (SUTAQ *Enhanced Care* subscale), and 159 out of 190 (83.7%) found it rewarding and worthwhile (UES *Rewarding* subscale). Postintervention interviews provided further insights into how perceiving benefits—from learning new tools and techniques to observing improvements in symptoms—was key to maintaining engagement, consistent with theory identifying perceived efficacy as a core construct influencing acceptability [20,21]. While prior research on conversational agent–led DMHIs has emphasized the role of factors such as empathy and human-likeness in fostering engagement [19], our findings underscore that expected outcomes and perceived efficacy remain central for individuals using these programs.

### Limitations

Limitations of this study are a lack of sample diversity and potential biases arising from the limited perspectives of the clinical and research teams. The program design and interpretation of qualitative data were shaped by the team’s cultural context. Given mental health inequities [64] and varying attitudes toward AI [65], research on DMHI acceptability in diverse populations is essential but remains limited [19,66]. Dedicated research is also needed on the personalization and adaptation of conversational agent–led DMHIs to user background, identity, culture, and socioeconomic context [67], as well as on the potential and limitations of generative AI in supporting such adaptation—for example, through language translation and tailoring, metaphor usage, or individualized treatment goals [68]. Co-production with diverse clinicians, researchers, and users with lived experience must be prioritized.

Quantitative findings may be biased toward participants who adhered to the full study protocol. For example, those who completed postintervention questionnaires were more likely to reach the minimum meaningful program dose within 9 weeks (168/203, 82.8%) compared with the full enrolled sample (180/300, 60%). The interviewed subsample, selected preintervention, showed similar engagement to the full sample (14/21, 67%), while those who completed postintervention interviews demonstrated higher engagement (12/16, 75%). Exploring participants’ expectations before the study helped mitigate this limitation and provided insights into factors important for acceptability, regardless of engagement. Nonetheless, findings primarily reflect the perspectives of the most engaged participants. Actionable guidance for DMHI development should therefore focus on enhancing engagement across the broader population. Further research with nonengagers is essential to understand barriers to DMHI acceptability.

Moreover, there are limitations to the validity of the self-reported measures (UES, SUS, and SUTAQ) in this context. Engagement with DMHIs involves different motivations and behavioral processes among users experiencing mental ill health compared with the general digital systems or telehealth services on which these measures were developed and validated, making interpretability in this context challenging [7]. For example, the UES *Focused Attention* subscale (reflecting absorption in the experience) may be less applicable in a DMHI than in a noninterventional system, as suggested by the qualitative analysis, where participants described the program as demanding but rewarding. Additionally, the interpretability of the scales was constrained by the scarcity of suitable benchmarks. To address this, we reported item-level response data to aid interpretation, and qualitative findings provided deeper contextual insights.

### Implications for Future Research and Implementation

This study offers insights to inform future research and implementation efforts aimed at enhancing the acceptability, engagement, and usability of conversational agent–led DMHIs. Key areas of focus are balancing user autonomy with structure and guidance, identifying when human support is most beneficial, and reflecting progress back to users to strengthen perceived efficacy and engagement. Real-world implementation pilots will be important to maximize adoption and outcomes [69,70]. The variability in user preferences across these domains underscores the need to explore personalized solutions. Advances in natural language generation using large language models offer powerful tools for creating varied, personalized interactions that surpass the limitations of rule-based dialogue systems [58]. However, their implementation must be approached with caution, ensuring outputs are guided by evidence-based protocols and safeguarded against inappropriate or potentially harmful content. Given users’ parallel needs for both structure and effectiveness, future DMHIs should explore safe and engaging ways to integrate generative AI within structured, evidence-based programs that incorporate feedback from lived experience. Finally, the study highlights the need to develop validated measures that capture the unique dimensions of user experience in conversational agent–led DMHIs, enabling more accurate quantitative evaluations of acceptability and engagement and facilitating further research into their relationship with clinical outcomes.

### Conclusions

This study provides evidence for the acceptability of a structured, conversational agent–driven digital program with human support for adults experiencing symptoms of generalized anxiety, along with insights to optimize such interventions. Understanding patient acceptability through multiple methods is crucial for enhancing engagement with DMHIs, a key challenge limiting their real-world impact. The findings underscore the importance of design, clinical, and implementation factors in supporting adherence. Importantly, patient expectations of conversational agents are rapidly shifting with the widespread availability of generative AI tools such as ChatGPT. Generative AI has the potential to transform the user experience with DMHIs by enabling dynamic, empathetic, and personalized interactions across modalities and languages. However, rigorous evaluation is essential to ensure patient safety and equitable health outcomes, alongside continued education and research into its acceptability. If integrated safely within structured, evidence-based, and effective DMHIs—shown here to be acceptable to users—this technology could revolutionize the delivery of mental health care and improve patient outcomes globally.

## Appendix

Multimedia Appendix 1 Additional analysis methods.

Multimedia Appendix 2 Self-reported measures of user experience.

## Abbreviations

AI: artificial intelligence
CBT: cognitive behavioral therapy
CONSORT: Consolidated Standards of Reporting Trials
DMHI: digital mental health intervention
GAD-7: 7-item Generalized Anxiety Disorder
ICD-10: International Classification of Diseases, 10th Revision
NHS: National Health Service
PHQ-9: 9-item Patient Health Questionnaire
PPI: patient and public involvement
SUS: System Usability Scale
SUTAQ: Service User Technology Acceptability Questionnaire
UES: User Engagement Scale
UKCA: UK Conformity Assessed

## Data Availability

Owing to the potential risk of patient identification, and following data privacy policies at ieso, individual-level data are not available. Aggregated data are available upon request, subject to a data-sharing agreement with ieso. Data requests should be sent to the corresponding author (PP) and will be responded to within 30 days.
