# Supplementary material for: Acceptability of a Conversational Agent–Led Digital Program for Anxiety: Mixed Methods Study of User Perspectives
Source: JMIR Hum Factors. 2025 Nov 4;12:e76377. doi: 10.2196/76377 (PMC12627969; doi:10.2196/76377)
Supplement: Multimedia Appendix 1 [file humanfactors_v12i1e76377_app1.docx]

## Multimedia Appendix 1 – Supplementary Methods

### A. Semi-structured interview schedules

#### Pre-intervention interviews

**Section 1 – Motivation**

1. What was your motivation to sign up to the study?
2. Do you think that this program is the right solution for you at this time?

**Section 2 – Views and previous experiences with different mental health support**

1. How do you think technology can help you improve your mental health?
2. Have you tried any other digital tools for your mental health before? *If yes:*
   1. How did you find it? What was helpful and what was not helpful?
   2. How different do you think the ieso Digital Program will be from the tools you used?
3. Is it your first time accessing any type of mental health support? *If no:*
   1. What support / therapy have you accessed before? (e.g. face-to-face therapy, CBT, counselling, etc.)
   2. How different do you think the ieso Digital Program will be from what you have done before?
4. What was your journey in seeking mental health support?
   1. Did you have any setbacks?

**Section 3 – Expectations for the program**

1. Based on what you know of the ieso Digital Program, how do you imagine it to be?
2. Can you tell me about any doubts you might have about doing this program?
3. Do you believe that this program can reduce your symptoms of anxiety? Why or why not?
4. How do you think this program will fit into your lifestyle?
5. What do you think will be the main benefits of using the ieso Digital Program?
6. Can you tell me about any concerns you might have about the ieso Digital Program?
7. Which of your needs do you think the ieso Digital Program will meet?
8. Which of your needs do you think the ieso Digital Program will not meet?
9. What are your expectations about interacting with a digital guide in the app?
10. What are your views on using an AI-powered digital guide in mental health care?
11. What are your expectations about human contact during the program?

#### Post-intervention interviews

**Section 1 – Overall experience of the ieso Digital Program**

1. Tell me about your experience with the program, how was it for you?
2. How did the program meet your expectation? How did it not meet your expectations?
3. What did you find most valuable or impactful in addressing your anxiety?
4. What did you find most frustrating about the program?

**Section 2 – Perceived Fit**

1. Do you feel that this program is well suited for people in your situation? (prompt: e.g. your age, gender, lifestyle, mental health experience, etc.)
2. Are there any specific groups of people that you think this program would be well-suited or most helpful for?

**Section 3 – Perceived support and safety**

1. How supported did you feel while using the program?
2. How safe did you feel while using the program? Was the support enough to help you feel safe?
3. Tell me about your experience with the fortnightly calls with the research coordinators? *Prompts*:
   1. How helpful or unhelpful were they?
   2. How often do you think these should be?
   3. How long do you think these should be?
4. Was there any point during the study where you felt worse? *If yes:*
   1. Did you think this was related to using the app?
   2. Did you think this was related to other parts of the study outside of the app? (e.g. assessments, calls, messages with your clinician…)
   3. Did you know what to do or who to contact in this situation?

**Section 4 – Program and app design**

1. What did you think of the length of sessions and of the overall program?
2. What did you think of the frequency of sessions and activities?
3. How did this program fit into your schedule?
4. What helped you or made you want to continue with the program? E.g. the app, the program, other…
5. What did you think of the reminders in the app?
6. How did you find the interaction with the digital guide through the sessions? *Prompts:*
   1. How well or poorly did you feel the digital guide listened and responded to your entries?
   2. Tell me some instances where you felt the digital guide could be more helpful?
7. Can you reflect on if and how you practiced the tools and techniques you learned in the sessions?
   1. Did you practice them during your personal time between sessions?
   2. What helped you practice these techniques?
   3. What were the barriers to practicing these techniques?
   4. What do you think the app could do to encourage and better support you in engaging between sessions in your own time?

**Section 5 – Perceived effectiveness**

1. Can you share any specific examples of techniques you found beneficial in your daily life>
2. How motivated did you feel to make changes after using the program?
3. How effective do you believe this program is?
   1. How effective do you think it is relative to in-person therapy (or other treatments or support you received in the past)

**Section 6 – Closing questions**

1. If you had a magic wand and could build your own program to help individuals with anxiety and/or depression, what is the one thing you would be sure to include?
2. Is there anything else you would like to share or ask?

### B. Qualitative Analysis Details

| **Analytical step** | | **Details (process, meetings, decisions and developments)** |
| --- | --- | --- |
| 1 | Familiarization | P.P. familiarized with the data by reviewing interview recordings and reading interview transcripts. Analytical and reflexive notes were written to document the process and discuss with the team at later stages. |
| 2 | Coding | P.P. inductively coded the interview transcripts line-by-line in Dovetail. Semantic codes were used to capture explicit meaning in the data, and latent codes were used to capture implicit meaning. Sections could be double coded when including multiple meanings and insights. P.P. met with research team members trained in qualitative methods and user research methods (M.B, M.Z.) to discuss codes and patterns in the data. Frequent codes were presented, coded extracts were reviewed, and interpretations discussed. |
| 3 | Candidate themes | After all pre-intervention and post-intervention interviews were coded, P.P. organized codes into candidate themes using mind mapping software, to identify patterns relevant to user engagement, acceptability and usability of the program. P.P. met with research team members (M.B., M.Z.) to present and discuss candidate themes to answer our research questions. |
| 4 | Refining themes | P.P. refined themes and sub-themes by reviewing the full dataset (re-reading transcripts) to ensure accurate representation of the data while focusing on the topics of interest to answer our research questions.  P.P. presented the first iteration of reviewed themes to the wider study team (M.Z., E.M., M.B., E.C., A.C., C.P.). Content of themes were described in detail with illustrative quotes. Group discussions highlighted that findings were in line with findings from other data collected through feedback surveys and helped make decisions to improve the clarity and cohesiveness of themes: sub-themes were moved or brought together, and theme titles were improved as a group.  P.P. presented the second iteration of reviewed themes to individuals with lived experience of mental health in a workshop. Content of themes and illustrative quotes were presented. The following types of feedback were requested: general feedback and understanding of themes, thoughts on theme titles, clarity and delineation of themes. All feedback and discussion points were recorded. |
| 5 | Defining final themes | Final themes were defined by P.P and C.P. by clarifying thematic boundaries, confirming theme titles and writing theme synopses and a thematic map. |
| 6 | Framework method | Inductive themes from the thematic analysis were used to create an analytical framework on Excel, and data was summarized for each participant by sub-theme, theme and interview time (pre- and post-), with illustrative quotes. Pre-and post- data was reviewed for each participant and across participants to explore changes in user perspectives pre- and post-intervention. |
| 7 | Write-up | Theme order and content was written and organized to provide the best narrative answer to our research question. Data extracts were chosen based on their vividness for the theme and representation of the overall data, and insights from the framework method were added where relevant to theme descriptions. |
