# Supplementary material for: Acceptability of a Conversational Agent–Led Digital Program for Anxiety: Mixed Methods Study of User Perspectives
Source: JMIR Hum Factors. 2025 Nov 4;12:e76377. doi: 10.2196/76377 (PMC12627969; doi:10.2196/76377)
Supplement: Multimedia Appendix 2 [file humanfactors_v12i1e76377_app2.docx]

## Multimedia Appendix 2 – Supplementary tables (Self-reported measures of user experience)

### User Engagement Scale (UES) item-level results

| **Supplementary table 1** – Raw UES item level agreement data in the whole sample (N=190) | | | | | | |
| --- | --- | --- | --- | --- | --- | --- |
| Subscale | Items | Proportion agreement N (%) | | | | |
|  |  | Strongly disagree | Disagree | Neither agree nor disagree | Agree | Strongly agree |
| **Focused attention** | I lost myself in this experience. | 24 (13) | 69 (36) | 59 (31) | 33 (17) | 5 (3) |
|  | The time I spent using ieso's digital programme just slipped away. | 9 (5) | 40 (21) | 58 (31) | 66 (35) | 17 (9) |
|  | I was absorbed in this experience. | 4 (2) | 41 (22) | 50 (26) | 77 (41) | 18 (9) |
| **Perceived Usability ^a^** | I felt frustrated while using ieso's digital programme. | 33 (17) | 64 (34) | 26 (14) | 49 (26) | 18 (9) |
|  | I found ieso's digital programme confusing to use. | 86 (45) | 80 (42) | 16 (8) | 6 (3) | 2 (1) |
|  | Using ieso's digital programme was taxing. | 37 (19) | 87 (46) | 35 (18) | 26 (14) | 5 (3) |
| **Aesthetic Appeal** | ieso's digital programme was attractive. | 2 (1) | 6 (3) | 60 (32) | 90 (47) | 32 (17) |
|  | ieso's digital programme was aesthetically appealing. | 2 (1) | 8 (4) | 31 (16) | 108 (57) | 41 (22) |
|  | ieso's digital programme appealed to my senses. | 3 (2) | 12 (6) | 47 (25) | 97 (51) | 31 (16) |
| **Rewarding** | ieso's digital programme was worthwhile. | 4 (2) | 5 (3) | 18 (9) | 93 (49) | 70 (37) |
|  | My experience was rewarding. | 5 (3) | 10 (5) | 32 (17) | 92 (48) | 51 (27) |
|  | I felt interested in this experience. | 2 (1) | 6 (3) | 10 (5) | 108 (57) | 64 (34) |
| ^a^ Items are reverse scored to produce the subscale scores in table 2 | | | | | | |
|  | | | | | | |

### System Usability Scale (SUS) item-level results

| **Supplementary table 2** – Raw SUS item level agreement data in the whole sample (N=203) | | | | | |
| --- | --- | --- | --- | --- | --- |
| Items | Proportion agreement N (%) | | | | |
|  | Strongly disagree | Disagree | Neither agree nor disagree | Agree | Strongly agree |
| I think that I would like to use this system frequently | 9 (4.4) | 31 (15.3) | 28 (13.8) | 97 (47. 8) | 38 (18.7) |
| I found the system unnecessarily complex | 84 (41.4) | 94 (46.3) | 15 (7.4) | 8 (3.9) | 2 (1.0) |
| I thought the system was easy to use | 1 (0.5) | 11 (5.4) | 12 (5.9) | 97 (47. 8) | 82 (40.4) |
| I think that I would need the support of a technical person to be able to use this system | 125 (61.6) | 63 (31.0) | 11 (5.4) | 2 (1.0) | 2 (1.0) |
| I found the various functions in this system were well integrated | 5 (2.5) | 12 (5.9) | 30 (14. 8) | 110 (54.2) | 46 (22.7) |
| I thought there was too much inconsistency in this system | 64 (31.5) | 80 (39.4) | 34 (16.8) | 21 (10.3) | 4 (2.0) |
| I would imagine that most people would learn to use this system very quickly | 0 (0) | 4 (2.0) | 14 (6.9) | 113 (55.7) | 72 (35.5) |
| I found the system very cumbersome to use | 74 (36. 5) | 89 (43.8) | 26 (12.8) | 9 (4.4) | 5 (2.5) |
| I felt very confident using the system | 1 (0.5) | 3 (1.5) | 19 (9.4) | 93 (45.8) | 87 (42.9) |
| I needed to learn a lot of things before I could get going with this system | 120 (59.1) | 69 (34.0) | 8 (3.9) | 6 (3.0) | 0 (0) |

### Service User Technology Acceptability Questionnaire (SUTAQ) item-level results

| **Supplementary table 3** – Raw SUTAQ item level agreement data in the whole sample (N=203) | | | | | | | | |
| --- | --- | --- | --- | --- | --- | --- | --- | --- |
| Subscale | Items ^a^ | | Proportion agreement N (%) | | | | | |
|  |  |  | Strongly  disagree | Moderately  disagree | Mildly  disagree | Mildly  agree | Moderately  agree | Strongly  agree |
| **Care personnel concerns** | *9* | I am concerned about the level of expertise of the individuals who monitor my status via the digital programme. | 98 (48.3) | 62 (30.5) | 22 (10.8) | 12 (5.9) | 7 (3.5) | 2 (1.0) |
|  | *20* | The digital programme interferes with the continuity of the care I receive (i.e. I do not see the same care professional each time). | 76 (37.4) | 53 (26.1) | 52 (25.6) | 18 (8.9) | 3 (1.5) | 1 (0.5) |
|  | *21* | I am concerned that the person who monitors my status, through the digital programme, does not know my personal health/social care history. | 46 (22.7) | 52 (25.6) | 42 (20.7) | 38 (18.7) | 20 (9.9) | 5 (2.5) |
| **Enhanced care** | *10* | The digital programme has allowed me to be less concerned about my health and/or social care. | 15 (7.4) | 19 (9.4) | 38 (18.7) | 61 (30.1) | 51 (25.1) | 19 (9.4) |
|  | *11* | The digital programme has made me more actively involved in my health. | 5 (2.5) | 7 (3.5) | 5 (2.5) | 58 (28.6) | 70 (34.5) | 58 (28.6) |
|  | *13* | The digital programme allows the people looking after me, to better monitor me and my condition. | 19 (9.4) | 15 (7.4) | 27 (13.3) | 70 (34.5) | 42 (20.7) | 30 (14.8) |
|  | *15* | The digital programme can be/should be recommended to people in a similar condition to mine. | 6 (3.0) | 9 (4.4) | 16 (7.9) | 36 (17.7) | 48 (23.7) | 88 (43.4) |
|  | *17* | The digital programme can certainly be a good addition to my regular health or social care. | 4 (2.0) | 3 (1.5) | 6 (3.0) | 32 (15.8) | 48 (23.7) | 110 (54.2) |
| **Increased accessibility** | *1* | The digital programme has saved me time in that I did not have to visit my GP clinic or other health/social care professional as often. | 16 (7.9) | 11 (5.4) | 21 (10.3) | 44 (21. 7) | 34 (16.8) | 77 (37.9) |
|  | *3* | The digital programme has increased my access to care (health and/or social care professionals). | 14 (6.9) | 18 (8.9) | 27 (13.3) | 68 (33.5) | 45 (22.2) | 31 (15.3) |
|  | *4* | The digital programme has helped me to improve my health. | 6 (3.0) | 3 (1.5) | 12 (5.9) | 62 (30.5) | 67 (33.0) | 53 (26.1) |
|  | *19* | The digital programme has made it easier to get in touch with health and social care professionals. | 11 (5.4) | 20 (9.9) | 44 (21.7) | 54 (26.6) | 42 (20.7) | 32 (15.8) |
| **Kit as substitution** | *16* | The digital programme can be a replacement for my regular health or social care. | 33 (16.3) | 44 (21.7) | 42 (20.7) | 43 (21.2) | 31 (15.3) | 10 (4.9) |
|  | *18* | The digital programme is not as suitable as regular face to face consultations with the people looking after me. ^b^ | 12 (5.9) | 22 (10.8) | 36 (17.7) | 51 (25.1) | 40 (19.7) | 42 (20.7) |
|  | *22* | The digital programme has allowed me to be less concerned about my health status. | 15 (7.4) | 19 (9.4) | 35 (17.2) | 68 (33.5) | 42 (20.7) | 24 (11.8) |
| **Privacy & discomfort** | *2* | The digital programme has interfered with my everyday routine. | 62 (30.5) | 47 (23.2) | 44 (21.7) | 41 (20.2) | 7 (3.5) | 2 (1.0) |
|  | *5* | The digital programme has invaded my privacy. | 125 (61.6) | 51 (25.1) | 19 (9.4) | 5 (2.5) | 3 (1.5) | 0 (0) |
|  | *8* | The digital programme has made me feel uncomfortable, e.g. physically or emotionally. | 82 (40.4) | 43 (21.2) | 36 (17.7) | 29 (14.3) | 8 (3.9) | 5 (2.5) |
|  | *12* | The digital programme makes me worried about the confidentiality of the private information being exchanged through it. | 98 (48.3) | 52 (25.6) | 29 (14.3) | 13 (6.4) | 9 (4.4) | 2 (1.0) |
| **Satisfaction** | *6* | The digital programme has been explained to me sufficiently. | 1 (0.5) | 2 (1.0) | 5 (2.5) | 17 (8.4) | 47 (23.2) | 131 (64.5) |
|  | *7* | The digital programme can be trusted to work appropriately. | 8 (3.9) | 6 (3.0) | 19 (9.4) | 44 (21.7) | 79 (38.9) | 47 (23.2) |
|  | *14* | I am satisfied with the digital programme I received. | 7 (3.5) | 9 (4.4) | 13 (6.4) | 38 (18.7) | 65 (32.0) | 71 (35.0) |
| ^a^ Items are preceded by a number representing the order in which the item was presented to participants.  ^b^ Items are reverse scored to produce the subscale scores in table 2 | | | | | | | | |
